# Electrochemical Synthesis of Ammonia: Recent Efforts and Future Outlook

**DOI:** 10.3390/membranes9090112

**Published:** 2019-08-30

**Authors:** Ioannis Garagounis, Anastasios Vourros, Demetrios Stoukides, Dionisios Dasopoulos, Michael Stoukides

**Affiliations:** 1 Department of Chemical Engineering, Aristotle University, 54124 Thessaloniki, Greece; 2 Chemical Processes & Energy Resources Institute, Center for Research and Technology Hellas, 56071 Thessaloniki, Greece

**Keywords:** ammonia synthesis, electrochemistry, solid state ammonia synthesis, molten electrolytes, aqueous electrolytes, non-aqueous electrolytes

## Abstract

Ammonia is a key chemical produced in huge quantities worldwide. Its primary industrial production is via the Haber-Bosch method; a process requiring high temperatures and pressures, and consuming large amounts of energy. In the past two decades, several alternatives to the existing process have been proposed, including the electrochemical synthesis. The present paper reviews literature concerning this approach and the experimental research carried out in aqueous, molten salt, or solid electrolyte cells, over the past three years. The electrochemical systems are grouped, described, and discussed according to the operating temperature, which is determined by the electrolyte used, and their performance is valuated. The problems which need to be addressed further in order to scale-up the electrochemical synthesis of ammonia to the industrial level are examined.

## 1. Introduction

The first ammonia plant started its operation in September 1913 and produced 5 tons/day [1,2]. In the past hundred years, the production of ammonia gradually increased to almost 200 million tons/year [3,4]. Nearly 80% of this is used in the fertilizer industry. Clearly, ammonia synthesis from its elements was of crucial importance to the growth of agriculture worldwide and consequently, for maintaining the growth of human population [1,2,3,4,5]. In addition to its use in agriculture, ammonia is expected to play a key role in modern, environmentally benign, energy technologies. Hydrogen is considered the energy currency of the future. Ammonia is a carbon-free molecule and hosts three hydrogen atoms. Hence, it is an excellent hydrogen storage compound. Furthermore, its decomposition produces high purity hydrogen, suitable to use in fuel cells [6,7,8].

The dominant process for ammonia synthesis is the Haber-Bosch (H-B) process which involves the production of H_2_ from the steam-reforming of natural gas, or coals, which concurrently produces enormous amounts of CO_2_. This is followed by extensive purification of this H_2_, before its reaction with N_2_ at 400–500 °C and at elevated pressures (about 150 bar) over a Fe-based catalyst [1,2]. Ever since its discovery, the H-B process has been gradually improved upon. The improvements consisted primarily in searching for more active catalysts which would allow operation at lower pressures and temperatures.

**Figure 1:**
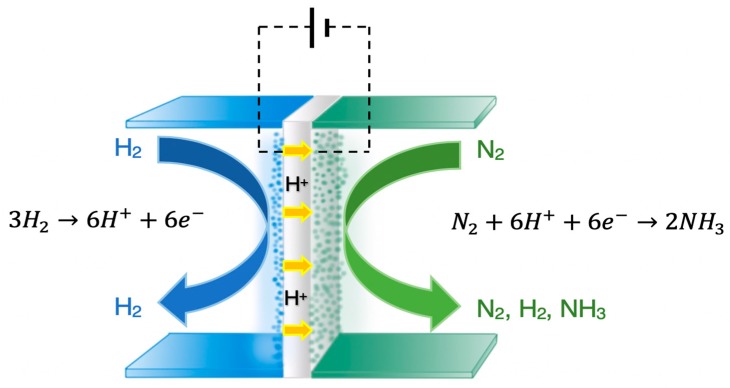
Schematic diagram of a solid state H^+^ conducting cell where NH_3_ is produced from gaseous H_2_ and N_2_.

In addition to the catalyst optimization, alternative routes to ammonia synthesis have been examined in the past three decades, including biocatalysis, photocatalysis, and electrocatalysis [9]. The latter has been studied extensively after the discovery of solid state proton (H^+^) conductors, i.e., materials that exhibit protonic conductivity at high temperatures (>400 °C), by H. Iwahara and his co-workers [10]. An electrochemical cell that can operate at high temperatures is advantageous compared to a low-temperature (aqueous) cell, since much higher reaction rates can be obtained on the same electrode area, as well as avoiding the formation of hydrazine. In 1998 [11], ammonia was synthesized from its elements at atmospheric pressure in the solid state H^+^ cell schematically shown in Figure 1. Gaseous H_2_ and N_2_ were introduced over the anode and the cathode, respectively. At the anode, H_2_ was converted into protons, which were electrochemically transferred to the cathode and reacted with N_2_ to produce NH_3_:

Anode: 3H_2_ → 6H^+^ + 6e^−^ (1)

Cathode: N_2_ + 6H^+^ + 6e^−^ → 2NH_3_ (2)

Overall: N_2_ + 3H_2_ ⇄ 2NH_3_ (3)

**Figure 2:**
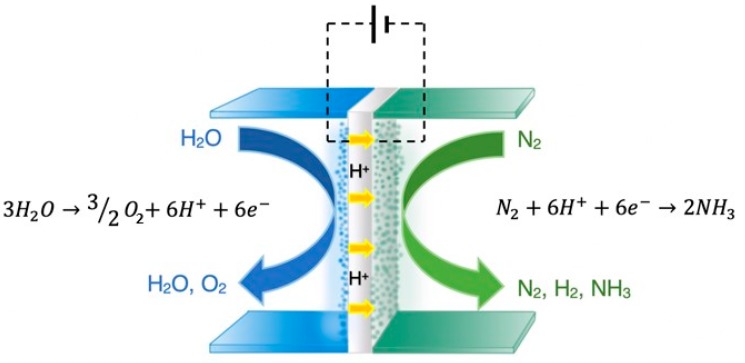
Schematic diagram of a solid state H^+^ conducting cell where NH_3_ is produced from H_2_O (steam) and N_2_.

In an effort to minimize material and energy consumption, several research groups have tested this approach (named Solid State Ammonia Synthesis, SSAS) in the past 20 years [1,5,7,12]. In 2009 [10], SSAS was experimentally demonstrated in the solid electrolyte cell of Figure 2, in which steam was used as a hydrogen source.

In this work, the anodic, cathodic, and overall reactions are

Anode: 3H_2_O → 6H^+^ + 6e^−^ + 3/2O_2_ (4)

Cathode: N_2_ + 6H^+^ + 6e^−^ → 2NH_3_ (5)

Overall: N_2_ + 3H_2_O ⇄ 2NH_3_ + 3/2O_2_ (6)

The promising results of these SSAS works motivated a large number of research groups to study the electrochemical synthesis of ammonia, not only at high, but also at moderate (150–350 °C) and low (<100 °C) temperatures. Recent review articles [1,3,4,5] have discussed and evaluated the most important findings from the works published from 2011 to 2015. The above reviews identified the problems that should be solved in order to promote the electrochemical synthesis of ammonia into industrial practice, which can be summarized as (a) low catalytic activity of the cathodic electrode (and consequently, reduced Faradaic Efficiency) and (b) low protonic conductivity of the cell. 

In order to address and solve the above problems, a large number of studies have been reported after 2016, almost as many as in all the previous years. The present review discusses the progress that has been achieved since then. The most promising results are presented and discussed. The future outlook of this electrochemical approach is also presented.

## 2. Recent Experimental Findings

### 2.1. High Temperature Studies

Table 1 contains the reported results on the electrochemical synthesis of ammonia at elevated (T > 100 °C) temperatures, from 2016 to 2018. The first column shows the operating temperature of each work. The second, third, and fourth columns contain the type of cathode (catalyst), anode, and electrolyte used in each study. The fifth column gives the (maximum) reaction rate attained, r_NH3_, expressed in moles of NH_3_ produced per second and per cm^2^ of electrode area. The sixth column shows the (maximum) percent Faradaic Efficiency, (FE), which is defined as the fraction of H^+^ that reacts with N_2_ to form NH_3_. In most studies, FE is limited because of the competing reaction of hydrogen evolution. The reference of each study is given in the last column. 

**Figure 3:**
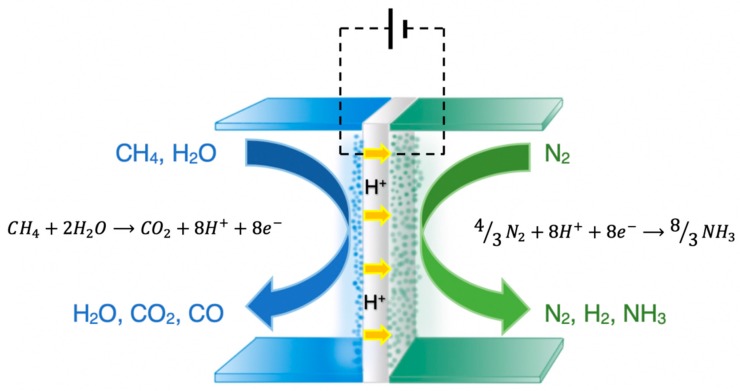
Schematic diagram of the electrochemical membrane reactor used for NH_3_ synthesis from CH_4_, H_2_O, and N_2_.

As shown in Table 1, the “high” temperature studies were conducted at temperatures between 200 and 650 °C. The highest reaction rate (8.27 × 10^−9^ mol·s^−1^·cm^−2^) was reported by Cui et al. [19] while the highest FE (14%) was reported by Kyriakou et al. [18]. Figure 3 schematically depicts the electrochemical Haber-Bosch process, proposed and tested in their work [18]. A methane-steam mixture is introduced at the anode (Ni-BZCY), while the cathode is exposed to gaseous N_2_. The following reactions take place over the anode under either open or under closed circuit.

CH_4_ + H_2_O ⇄ CO + 3H_2_ (7)

CO + H_2_O ⇄ CO_2_ + H_2_ (8)

Upon closing the circuit, however, the produced hydrogen is converted into H^+^ and the electrochemical reaction can be written as:

CH_4_ + 2H_2_O → CO_2_ + 8H^+^ + 8e^−^ (9)

The produced H^+^ are transferred to the cathode (VN-Fe) through the BaZr_0.8_Ce_0.1_Y_0.1_O_2.95_ (BZCY81) solid electrolyte, where they react with nitrogen towards ammonia:

4/3N_2_ + 8H^+^ + 8e^−^ → 8/3NH_3_ (10)

The total reaction of the cell of Figure 3 is derived from the sum of Reactions (9)–(10):

CH_4_ + 2H_2_O + 4/3N_2_ → CO_2_ + 8/3NH_3_ (11)

At 500–650 °C and upon imposition of constant currents, hydrogen is not only separated from the anode side, but simultaneously as much as 14% of it is converted to ammonia at the cathode at low cell potentials (< 0.4 V). The maximum rate of NH_3_ production was 1.9 × 10^−9^ mol·s^−1^·cm^−2^. At the same time, the conversion of CH_4_ reached 80% with an up to 96% selectivity to CO_2_ [18].

**Figure 4:**
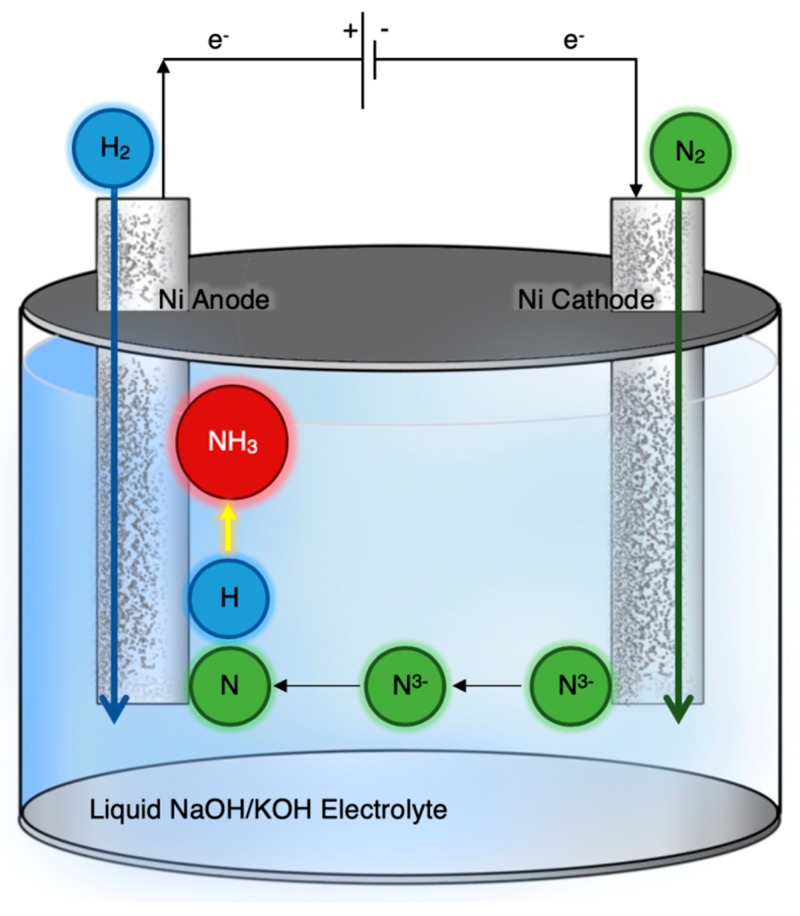
Schematic diagram of a molten salt (NaOH-KOH) cell where NH_3_ is produced from the reaction of H_2_ with N^3−^ ions. Redrawn from [20].

Figure 4 is a schematic diagram of the apparatus used by Y. Bicer and I. Dincer [20]. The electrolyte was a molten salt (NaOH-KOH) and two porous nickel mesh electrodes were used for anode and cathode. At the latter, N_2_ was converted to N^3−^ ions, which were transported to the anode and reacted with H_2_ to produce NH_3_. Experiments were conducted in the range of 200–255 °C with suspended Fe_3_O_4_ nanoparticles used as the catalyst. The optimum operating temperature was 200 °C, at which the ammonia production rate of 6.54 × 10^−10^ mol·s^−1^·cm^−2^ was obtained with a corresponding FE of 9.3% [20].

Kishira et al. [13] studied the electrochemical synthesis of ammonia in a solid electrolyte cell, similar to Figure 1 and Figure 2, in which composites of CsH_2_PO_4_ and SiP_2_O_7_ were used as the electrolyte. The anode was a Pt/C-loaded carbon paper while several materials were tested as cathodes: Pt/C-, Pt-Ru/C-, Ru/C-, Ru-, and Ag-Pd-loaded carbon paper. Experiments were conducted at 220 °C and atmospheric pressure. When H_2_ was used as the hydrogen source, reaction rates higher than 10^−10^ mol·s^−1^·cm^−2^ were obtained. When H_2_O (steam) was used instead of H_2_, an order of magnitude decrease in the reaction rate was observed, however, the highest current efficiencies remained similar regardless of the anode feed [13].

Following up on their earlier work [23], Cui et al. studied the synthesis of ammonia from water and nitrogen [19]. An iron-based catalyst supported on activated carbon (Fe_2_O_3_/AC) was used as an electrocatalyst in a molten hydroxide (NaOH–KOH) cell. Experiments were carried out at 250 °C and atmospheric pressure. According to the mechanism proposed by the authors, the Fe_2_O_3_ catalyst is electrochemically reduced to Fe:

Fe_2_O_3_ ⇄ 2Fe + 3/2O_2_ (12)

The reduced iron reacts with N_2_ and H_2_O to produce NH_3_:

2Fe + N_2_ + 3H_2_O ⇄ 2NH_3_ + Fe_2_O_3_ (13)

Reactions (12) and (13) combined, give the overall reaction of NH_3_ synthesis from H_2_O and N_2_, i.e., Reaction (6). The highest rate (8.27 × 10^−9^ mol·s^−1^·cm^−2^) was obtained at a voltage of 1.55 V with a corresponding current density of 49 mA·cm^−2^. The highest FE, obtained at 1.15 V and 11 mA·cm^−2^ was 13.7% [19]; less than a third that in their previous work but considerably more stable. 

The effect of Electrochemical Promotion [24,25] on the kinetics of ammonia synthesis was studied by Kosaka et al. [16] in a BaCe_0.9_Y_0.1_O_3_ (BCY) proton conducting solid electrolyte double chamber cell with Pt and Fe-BCY used as anodic and cathodic electrodes, respectively. In the temperature range of 500–650 °C it was found that when pure N_2_ was introduced at the cathode side, an insignificant rate of ammonia formation was observed under cathodic polarization. When the cathode feed contained 15% H_2_ in N_2_ balance, however, an up to 20-fold increase in the reaction rate was observed under similar polarization (610 °C, −1.5 V vs. OCV).

The electrochemical promotion of ammonia synthesis was also studied in a K-β″-Al_2_O_3_ solid electrolyte (K^+^ conductor) single chamber cell by Díez-Ramírez et al. [22]. The effect of potassium addition on the cathodic electrode (Co_3_Mo_3_N) was studied at 400–550 °C. The rate of ammonia synthesis exhibited a volcano-type behavior with the maximum around 1% of potassium per total moles of Co_3_Mo_3_N. Values of Λ [25] as high as 300 were obtained, the highest reported thus far in NH_3_ synthesis [22].

### 2.2. Low Temperature Studies

Results of the “low” (<100 °C) temperature studies are summarized in Table 2.

**Figure 5:**
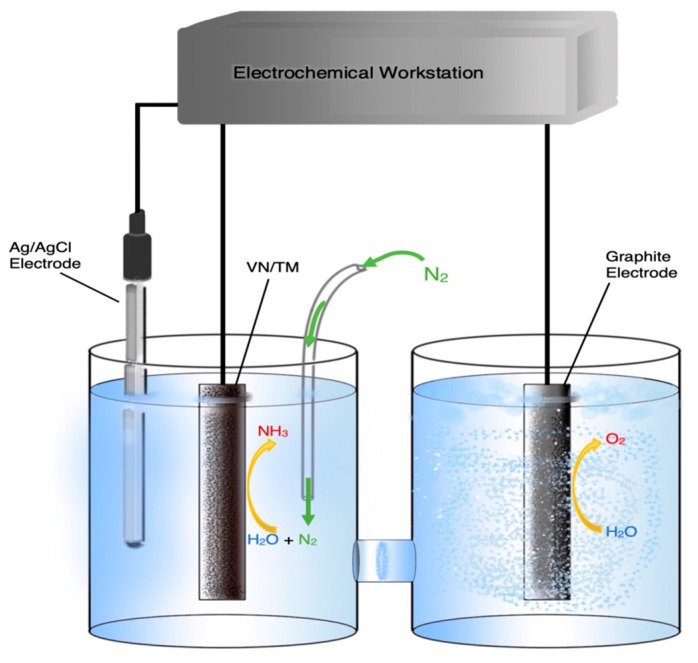
Schematic diagram of a HCl cell where NH_3_ is produced on a VN/(titanium mesh) (VN/TM) catalyst via a Mars-van Krevelen mechanism. Redrawn from [40].

The highest reaction rate was reported by Li et al. [44], while the highest FE (60%) was reported by Zhou et al. [55]. The former group prepared amorphous/low-crystalline Au nanoparticles and compared their reactivity towards NH_3_ synthesis to that of the crystalline counterpart [39]. The amorphous Au/CeO_x_ nanoparticles were anchored on reduced graphite oxide (RGO) to form the cathodic electrocatalyst in a setup similar to that of Figure 5. It was found that both ammonia yield and Faradaic efficiency were significantly higher when amorphous instead of crystalline particles were used. The maximum rate of NH_3_ synthesis was 2.7 × 10^−8^ mol·s^−1^·cm^−2^ and the highest FE attained was 10.1% [39]. This is the highest rate of all the works reported in the present review and, to our knowledge, one of the highest ever achieved in the electrochemical synthesis of NH_3_.

Another interesting work was published by Liu et al. who developed a new method for the synthesis of ultrathin, surfactant-free Rh nanostructures [50]. These Rh nanosheet nanoassemblies were used as electrocatalysts for ammonia synthesis in an ambient temperature and pressure cell similar to that of Figure 5, but with an alkaline electrolyte. They reported rates of NH_3_ production as high as 23.88 μg of NH_3_ per hour and per mg of catalyst, which corresponds to 6.24 × 10^−9^ mol·s·cm^−2^ [50]. However, they only achieved an FE of 0.7%, in complete contrast to Zhou et al. who reported FEs as high as 60% using hydrophobic ionic electrolytes with high nitrogen solubility and variously supported, nanostructured Fe cathodes [55]. On the other hand, the formation rates of the latter work were, at best, two orders of magnitude lower than those of the former [50]. 

In an effort to reduce the extent of the undesirable formation of H_2_ at the cathode, Zhang et al. used VN supported on a titanium mesh as a catalyst [40]. Previous theoretical and experimental works [58,59] suggested that VN is an effective ammonia synthesis catalyst as the reaction proceeds via a Mars-van Krevelen mechanism. According to the above mechanism, protons react with N atoms of the VN lattice and produce NH_3_, while gaseous N_2_ dissociates on the catalyst surface to refill the lattice vacancy. The advantage of the Mars-van Krevelen mechanism is that the VN catalyst participates in the reaction cycle and the rate determining step, i.e., the breaking of the N-N bond, essentially becomes a secondary or auxiliary process. In spite of these predictions, the highest rate and FE achieved by Zhang et al. in the cell shown schematically in Figure 5 were far from impressive at 8.40 × 10^−11^ mol·s^−1^·cm^2^ and 2.25%, respectively [40]. Another study using VN, this time supported on carbon cloth with an acidic electrolyte, reported similar inauspicious values of 2.48 × 10^−10^ mol·s^−1^·cm^−2^ and 3.58% [37].

**Figure 6:**
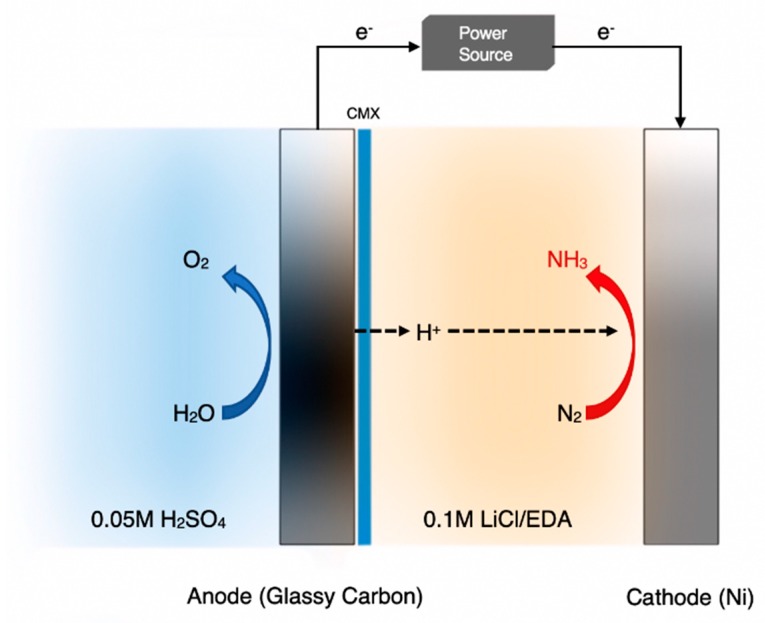
Schematic diagram of the ethylenediamine (EDA)-based cell for the electrochemical synthesis of NH_3_. Redrawn from [53].

A different solution to the same problem was attempted by Kim et al. [52,53] who exposed the cathode to non-aqueous media. First, they studied the reaction using 2-propanol as a cathodic solvent [52]. This, however, was unstable resulting in a poor FE (<1%). Consequently, the authors switched to the apparatus of Figure 6 [53], where the cathodic solvent was ethylenediamine (EDA). Thus, the cathode chamber was filled with 0.1 M LiCl/EDA and was separated from the anode chamber by a cation exchange membrane, while the anode was exposed to a 0.05 M H_2_SO_4_ aqueous solution. With respect to decreasing the evolution of hydrogen, this cell was very successful, compared to the previous attempt. Although the rate of NH_3_ formation was lower than 10^−10^ mol·s^−1^·cm^−2^, their Faradaic Efficiency reached as high as 17.2% at a cell voltage of 1.8 V [53].

**Figure 7:**
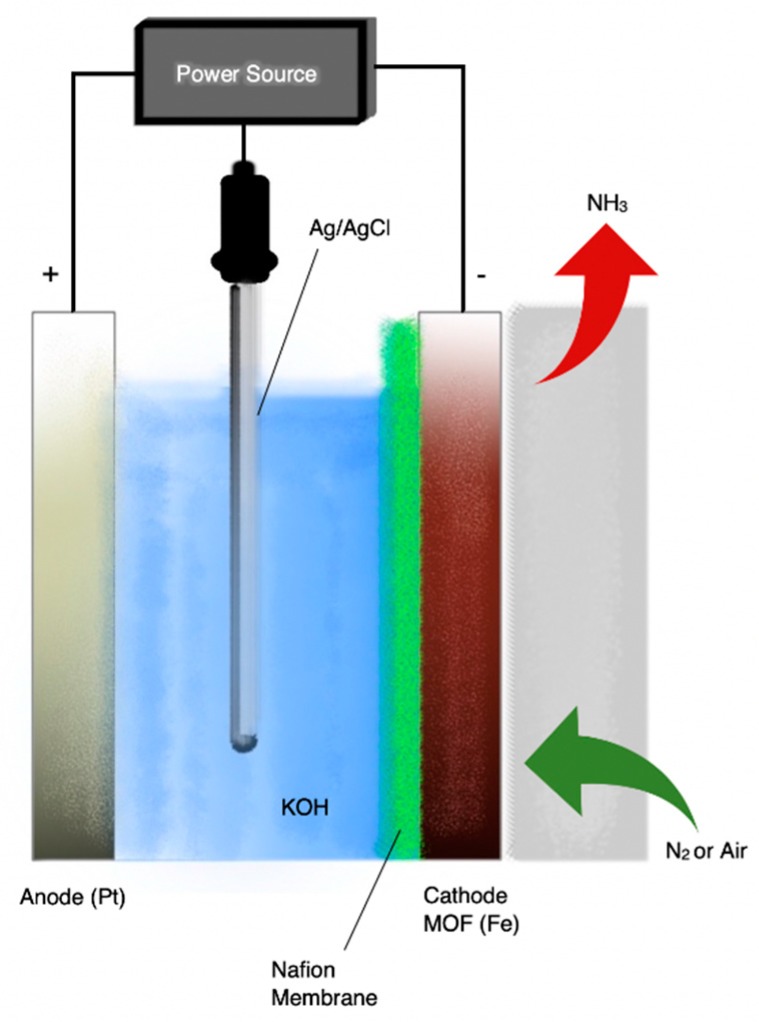
Schematic diagram of the KOH cell where NH_3_ production was catalyzed by metal–organic-frameworks (MOFs) of Fe, Cu, and Co. Redrawn from [49].

Figure 7 is a schematic diagram of the apparatus used by Zhao et al. [49]. The three-electrode cell used a 2 M KOH aqueous solution as the electrolyte-hydrogen source, a Nafion-117 membrane as the proton conductor and a Pt wire as anode. Metal-organic-frameworks (MOFs) were used as catalysts and were pressed together with a brass wire mesh to form the working electrode (cathode). The reactants were H_2_O and either N_2_ or air. The MOF(Fe) exhibited the highest catalytic activity MOF(Fe) with the ammonia formation rate and the current efficiency reaching 2.12 × 10^−9^ mol·s^−1^·cm^−2^ and 1.43%, respectively, when using pure N_2_ and H_2_O [49].

Yao et al. used surface-enhanced infrared absorption spectroscopy (SEIRAS) to investigate the mechanisms of nitrogen reduction on Au and Pt thin film supported on Si prisms, with a 0.1 M KOH electrolyte [60]. Their results indicated that the nitrogen reduction reaction on Au surfaces follows an associative mechanism, and the N≡N bond in N_2_ tends to break simultaneously with the hydrogen addition. By comparison, in their experiments no absorption band associated with N was observed on Pt surfaces under the same reaction condition [60].

Finally, the commendable effort of Shipman and Symes whose report refutes the previously published activity of Sn(II) phthalocyanine on carbon foil in a 1 M KOH solution [46] should be mentioned. The group found that substituting the N_2_ feed with Ar resulted in the same rate of NH_3_ formation, proving that the source of NH_3_ was not the NRR but the decomposition of the catalyst-electrode. This is important because it highlights the need for “blank” tests, especially when complex N-containing electrodes are used. Although such measurements are present in most of the recent studies, they are absent from many of the older ones and caution is necessary when citing such literature or publishing such works in future.

## 3. Discussion

A general observation from Table 2 is that studies reporting high faradaic efficiencies report very low formation rates, while those with higher rates have low FEs. In fact, of the studies in Table 2 almost all those with FE > 10% report rates below 10^−10^ mol·s^−1^·cm^−2^, with the most important exception being the work of Li et al. whose near record-breaking rate of 2.7 × 10^−8^ mol·s^−1^·cm^−2^ is obtained at a FE of 10.1% [39]. Another observation derived from comparing Table 1 with Table 2 is that high temperature studies report higher rates, on average by about an order of magnitude, but in spite of these higher rates, the same studies generally report lower FEs. This fact could be attributed to the decomposition of produced NH_3_, which becomes spontaneous above 175 °C and can be quite extensive at temperatures above 500 °C, depending on reactor geometry and residence times. Thus, in works carried out below 100 °C (Table 2) decomposition does not occur, but their main problem is the slow kinetics of the formation reaction. In this vein, it was proposed [4] that cells operating in the 200–250 °C range, e.g., with CsH_2_PO_4_ electrolytes, might be ideal for ammonia synthesis. The data of Table 1, however, show otherwise, with most such systems struggling to reach 10^−10^ mol·s^−1^·cm^−2^ and only three achieving FE > 1% [15,19,20]. While these underwhelming results might be attributed to the poor design and/or fabrication of the cathode or the electrode-electrolyte interphase, it may simply be that NRR kinetics at ambient pressure are still too slow, even at 250 °C.

A similarly large discrepancy between prediction and practice can be observed for nitride electrodes. While DFT calculations predicted FEs of, or close to, 100% for VN [58], the experimental studies report maximum values below 5% at ambient temperature [37,40] and not higher than 14% at 500–650 °C [18]. This difference could be attributed to the fact that the polycrystalline materials used in the latter works, possibly poorly interphased with the corresponding electrolytes, have little resemblance, on an adsorption/binding energy basis, to the much more rigidly ordered single-surface models of the former. This, in turn, can lead to heated and, likely, fruitless arguments over the practical merits of such modeling studies vs. the need for more advanced fabrication techniques.

Regarding the source of H^+^ used, all of the studies listed in Table 2 report water as the proton source, while the same is true for about half of those in Table 1. This is encouraging because the electrochemical process can run on renewable electricity with water, or steam, as the H^+^ source, thus avoiding the CO_2_-intensive hydrogen production step usual in conventional ammonia plants. The drawback in such systems is that the electricity demand, and more specifically the cell voltage, increases dramatically because of the high energy requirements of electrolysis [61]. However, almost all low temperature studies (Table 2) use a reference electrode and give voltage values for the cathode vs. RHE, which essentially hides the effect of the H+ source on the power consumption, since protons are produced at the anode. In order to calculate the electricity consumption of an ammonia cell one must use the potential difference between working and counter electrode (cathode and anode), since that is where the current flows. A different approach reported by Kyriakou et al. [18] employed a methane-steam mixture which produced hydrogen from methane reforming avoiding the high voltage requirements at the expense of chemical energy (the lower heating value of methane).

Finally, a note on conductivity, which has been identified in previous reviews as one of the main hurdles to overcome before practical applications [4]. In order to produce ammonia at high rates one must have an adequate supply of protons and, therefore, a high proton conductivity under the operating conditions (temperature, anode-cathode voltage and concentration/partial pressure of H^+^ source). However, a quick glance at Table 1 and Table 2 reveals that the main issue in most works is the Faradaic Efficiency, which is rarely higher than 10% and often well below 1%. It seems therefore imperative that suppressing hydrogen evolution at the cathode should be the first priority. Unfortunately, the more successful attempts in this direction [40,53,55] tend to compromise the conductivity and, thus, the highest FE of 60% corresponds to a rate of only 6.5 × 10^−12^ mol·s^−1^·cm^−2^ [55].

## 4. The Outlook

Previous reviews on the electrochemical synthesis of ammonia [1,2,3,4,5] had identified the problems that needed to be solved in order for this route to compete with the industrial H-B process. There were two main areas that needed improvement:

(a) The proton conductivity of the cells. Regardless of the hydrogen source, nitrogen must react with H^+^, which in turn, must be supplied electrochemically. The highest proton fluxes reported until the end of 2015 were of the order of 10^−7^ gram atoms of H^+^·s^−1^·cm^−2^ [4].
(b) The catalytic activity of the cathodic electrode. The reaction of hydrogen evolution competes with the reaction of NH_3_ synthesis and this results in a significant decrease in FE. Although FEs as high as 90% had been reported, in general the FE values are mostly lower than 10%. At temperatures <100 °C, most of the reported FE values are of the order 1% [1,4].

The experimental results of the past three years indicate a moderate progress. On the other hand, several alternatives to the “classic” electrochemical cell have been tested. The reason for this moderate success is that the proposed alternatives successfully solve one and not all the problems. For example, the works with molten salts [19,20] bypass the problem of hydrogen evolution at the cathode because the reaction of N^3−^ with H_2_ takes place at the anode (Figure 4). Nevertheless, the FE, which in this case is defined as the moles of NH_3_/s produced, divided by I/3F, was lower than 9.5% and 13.7% in [13] and [16], respectively. A significant increase in the FE, however, could be achieved upon improving the reactor design and the addition of more appropriate additives [20].

The use of metal nitrides and the synthesis of NH_3_ via a Mars-van Krevelen mechanism resulted in remarkably high reaction rates and FEs [18,37,40], but still well below the predictions by theoretical DFT calculations [58]. This disagreement between theory and practice is neither due to wrong calculations nor to experimental errors. In the case of Kyriakou et al., the cathodic electrode was not VN but VN-Fe (to improve adhesion with the ceramic electrolyte). In the presence of metallic Fe, the reaction of hydrogen evolution was enhanced and the obtained FEs were much lower than those predicted on pure single-crystalline VN [18,58].

The reaction of hydrogen evolution was successfully suppressed by using ionic liquids, instead of aqueous solutions, as electrolytes. Faradaic efficiencies as high as 60% were obtained [55]. Nevertheless, these high FE values were not accompanied by high NH_3_ yields. This is because the sole H^+^ source for the reaction was H_2_O, which in turn, had to remain at very low levels, i.e., of the order of 200 ppm.

The above summary indicates clearly that there is room for improving the performance of the NH_3_-producing electrochemical cells. On the other hand, an enhanced performance requires optimization in all key aspects, such as (a) selection of materials and construction of the cathodic electrode, (b) design and fabrication of the electrolyte-electrode interphase, and (c) selection of the operating conditions. The work of Kishira et al. [13] is an example of potential improvement. The electrochemical synthesis of ammonia in solid electrolyte cells operating at temperatures between 200 and 300 °C is expected to be advantageous because (a) the temperature is low enough to avoid NH_3_ decomposition as well as the difficulties in the construction of the cell (materials strength, durability, long term stability), (b) the temperature is high enough to obtain reasonably fast reaction kinetics. There were no SSAS studies at 200–300 °C because solid electrolytes with high conductivity and stability in this temperature regime were discovered and developed only recently [62,63,64,65]. Although the cell operated at the “ideal” temperature range, neither the reaction rate nor the FE were high (Table 1). In our opinion, the reason is that the selected operating temperature was the optimum but the other factors (cathode preparation, catalyst) were not.

In conclusion, the development and the scale up of the electrochemical synthesis of ammonia, although at a rather slow pace, is moving ahead. The reason for this slow progress is the need for intensive collaboration among scientists and engineers working in different fields (solid state ionics, catalysis, electrochemistry, reactor design) in order to bring this process into industrial practice. 

## Figures and Tables

**Table 1:** Studies at high temperatures.

Temp. (°C)	Cathode	Anode	Electrolyte	Reactants (Cathode/Anode)	r_NH3_ (mol∙s^−1^∙cm^−2^)	FE (%)	Ref.
220	Ru/C	Pt/C	CsH_2_PO_4_/SiP_2_O_7_ composite	N_2_/H_2_	8.5 × 10^−11^	0.075	[13]
220	Pt/C	Pt/C	CsH_2_PO_4_/SiP_2_O_7_ composite	N_2_/H_2_	2.3 × 10^−10^	0.05	[13]
220	Ru	Pt/C	CsH_2_PO_4_/SiP_2_O_7_ composite	N_2_/H_2_	1.7 × 10^−10^	0.12	[13]
220	Ag-Pd	Pt/C	CsH_2_PO_4_/SiP_2_O_7_ composite	N_2_/H_2_	8.5 × 10^−11^	0.1	[13]
220	Pt/C	Pt/C	CsH_2_PO_4_/SiP_2_O_7_ composite	N_2_/H_2_O	6.5 × 10^−12^	0.025	[13]
220	Pt-Ru/C	Pt/C	CsH_2_PO_4_/SiP_2_O_7_ composite	N_2_/H_2_O	1.3 × 10^−11^	0.04	[13]
220	Ru/C	Pt/C	CsH_2_PO_4_/SiP_2_O_7_ composite	N_2_/H_2_O	1.9 × 10^−11^	0.14	[13]
220	Ru	Pt/C	CsH_2_PO_4_/SiP_2_O_7_ composite	N_2_/H_2_O	1.25 × 10^−11^	0.055	[13]
220	Ag-Pd	Pt/C	CsH_2_PO_4_/SiP_2_O_7_ composite	N_2_/H_2_O	0.9 × 10^−11^	0.06	[13]
200–250	Ru/Cs^+^/MgO |Pd-Ag^*^	Pt	CsH_2_PO_4_/SiP_2_O_7_	N_2_/H_2_O	9 × 10^−10^	2.6	[14]
220	Pt/TiO_2_|C^*^	Pt/C	CsH_5_(PO_4_)_2_/SiO_2_	N_2_/H_2_O	2 × 10^−10^	2.1	[15]
500–650	K, Al modified Fe-BCY	Pt	BaCe_0.9_Y_0.1_O_3_ (BCY)	N_2_/H_2_	2.4 × 10^−11^	0.005	[16]
500–650	K, Al modified Fe-BCY	Pt	BaCe_0.9_Y_0.1_O_3_ (BCY)	N_2_-H_2_(15%)/H_2_	6.7 × 10^−10^	0.5	[16]
500	Ni-(BCYR) BaCe_0.8_Y_0.1_Ru_0.1_O_3_	Pt	BaCe_0.9_Y_0.1_O_3_ (BCY)	N_2_/H_2_O(2%)-H_2_(20%)	1.1 × 10^−11^	0.22	[17]
500	LST (La_0.3_Sr_0.6_TiO_3_)-BCYR	Pt	BaCe_0.9_Y_0.1_O_3_ (BCY)	N_2_/H_2_O(2%)-H_2_(20%)	1.1 × 10^−11^	2.1	[17]
500–650	VN-Fe	Ni-BZCY72	BZCY81	N_2_/CH_4_-H_2_O	1.89 × 10^−9^	14	[18]
250	Stainless steel (Fe_2_O_3_/AC)	Ni	NaOH-KOH molten salt with	N_2_/H_2_O	8.27 × 10^−9^	13.7	[19]
200–255	Ni (Fe_3_O_4_)	Ni	KOH-NaOH molten salt	N_2_/H_2_	6.54 × 10^−10^	9.46	[20]
327	Ni (Fe_2_O_3_)	Li-Al alloy	LiCl/KCl/CsCl	N_2_/H_2_O	3 × 10^−10^	N/A	[21]
327	Ni (CoFe_2_O_4_)	Li-Al alloy	LiCl/KCl/CsCl	N_2_/H_2_O	1.78 × 10^−10^	N/A	[21]
400–550	Co_3_Mo_3_N-Ag	Au	K-β″-Al_2_O_3_	N_2_/H_2_	2.7 × 10^−9^	Λ = 300	[22]

* Catalyst powder on top of |electrode.

**Table 2:** Studies at low temperatures.

Temp. (°C)	Cathode	Anode	Electrolyte	Reactants (Cathode/Anode)	r_NH3_ (mol∙s^−1^∙cm^−2^)	FE (%)	Ref
AT	Fe_2_O_3_/CP	Graphite rod	Nafion 211/0.1 M Na_2_SO_4_	N_2_/H_2_O	1.03 × 10^−10^	0.94	[26]
RT	MoS_2_/CC	Graphite rod	Nafion/0.1 M Na_2_SO_4_	N_2_/H_2_O	8.08 × 10^−11^	1.17	[27]
AT	Mo_2_C/C	Pt	Nafion 211/0.5 M Li_2_SO_4_	N_2_/H_2_O	N/A	7.8	[28]
25	PEBCD/CC	Pt	Nafion 211/0.5 M Li_2_SO_4_	N_2_/H_2_O	3.28 × 10^−11^	2.91	[29]
20	30% Fe_2_O_3_/CNT	Pt	Nafion 115/0.25 M K_2_SO_4_	N_2_/H_2_O	1 × 10^−11^	0.125	[30]
20	30% Fe_2_O_3_/CNT	Pt	Nafion 115/0.25 M KHSO_4_	N_2_/H_2_O	7.87 × 10^−12^	0.07	[30]
RT	NPC-750	Pt	Nafion 117/0.05 M H_2_SO_4_	N_2_/H_2_O	2.33 × 10^−10^	1.42	[31]
RT	Mo-D-R-5h	Pt	Membrane/0.01 M H_2_SO_4_	N_2_/H_2_O	3.09 × 10^−11^	0.72	[32]
AT	Pd/C	Pt	Nafion 115/0.05 M H_2_SO_4_	N_2_/H_2_O	1.2 × 10^−11^	0.03	[33]
RT	Au NPs/C_3_N_4_/CP	Pt	Nafion 115/0.5 M H_2_SO_4_	N_2_/H_2_O	N/A	6	[34]
RT	Au_1_/C_3_N_4_/CP	Pt	Nafion 115/0.5 M H_2_SO_4_	N_2_/H_2_O	N/A	11.1	[34]
RT	CP (Cp_2_TiCl_2_/[C_9_H_20_N]^+^ [(C_2_F_5_)_3_PF_3_]^-^)	Pt	Nafion 212/0.2 M H_2_SO_4_	N_2_/H_2_O	N/A	0.2	[35]
20	30% Fe_2_O_3_/CNT	Pt	Nafion 115/0.5 M KHCO_3_	N_2_/H_2_O	8.5 × 10^−12^	0.125	[30]
20	Fe_2_O_3_/CNT	Pt	Nafion/KHCO_3_	N_2_/H_2_O	3.59 × 10^−12^	0.15	[36]
RT	MoS_2_/CC	Graphite rod	Nafion/0.1 M HCl	N_2_/H_2_O	8.48 × 10^−11^	0.096	[27]
AT	VN/CC	Graphite rod	Membrane/0.1 M HCl	N_2_/H_2_O	2.48 × 10^−10^	3.58	[37]
60	Au/TiO_2_	Pt	Nafion 211/0.1 M HCl	N_2_/H_2_O	5 × 10^−10^	13.5	[38]
RT	Au/TiO_2_	Pt	Nafion 211/0.1 M HCl	N_2_/H_2_O	3.5 × 10^−10^	8.11	[38]
RT	Amorphous Au/CeOx-RGO	Pt	Nafion 211/0.1 M HCl	N_2_/H_2_O	2.7 × 10^−8^	10.1	[39]
RT	VN/(Titanium Mesh)	Graphite rod	Nafion/0.1 M HCl	N_2_/H_2_O	8.4 × 10^−11^	2.25	[40]
AT	B_4_C/CP	Graphite rod	Nafion 211/0.1 M HCl	N_2_/H_2_O	4.34 × 10^−11^	15.95	[41]
RT	Nb_2_O_5_/CP	Graphite rod	Membrane/0.1 M HCl	N_2_/H_2_O	6.8 × 10^−10^	9.26	[42]
AT	NCM	Pt	Membrane/0.1 M HCl	N_2_/H_2_O	1.3 × 10^−10^	5.2	[43]
AT	NCM-AuNPs	Pt	Membrane/0.1 M HCl	N_2_/H_2_O	5.88 × 10^−10^	22	[43]
AT	Pd/C	Pt	Nafion 115/0.1 M PBS	N_2_/H_2_O	2.2 × 10^−11^	8.2	[33]
AT	Au/C	Pt	Nafion 115/0.1 M PBS	N_2_/H_2_O	2.4 × 10^−12^	1.2	[33]
AT	Pt/C	Pt	Nafion 115/0.1 M PBS	N_2_/H_2_O	2.4 × 10^−12^	0.2	[33]
AT	Pd/C	Pt	Nafion 115/0.1 M NaOH	N_2_/H_2_O	1.07 × 10^−11^	0.075	[33]
AT	CoP (hollow nano-cages)	Pt	Nafion 117/1 M KOH	N_2_/H_2_O	8.8 × 10^−11^	7.36	[44]
AT	o-Fe_2_O_3_-CNT/CP	Graphite rod	Nafion/ 0.1 M KOH	N_2_/H_2_O	2.37 × 10^−11^	8.28	[45]
RT	Carbon foil (Sn(II) phthalocyanine)	Pt	1 M KOH	N_2_/H_2_O	1.4 × 10^−11^*	2*	[46]
RT	Tetrahexahedral Au/CP	Graphite plate	Nafion 211/0.1 M KOH	N_2_/H_2_O	2.7 × 10^−11^	3.9	[47]
65	Tetrahexahedral Au/CP	Graphite plate	Nafion 211/0.1 M KOH	N_2_/H_2_O	2.2 × 10^−10^	6.8	[47]
20	30% Fe_2_O_3_/CNT	Pt	Nafion 115/0.5 M KOH	N_2_/H_2_O	1.06 × 10^−11^	0.164	[30]
20	o-CNT	Pt	Nafion 115/0.5 M KOH	N_2_/H_2_O	3.44 × 10^−12^	-	[30]
65	Fe_2_O_3_/CP	Ti/IrO_2_	Membrane/0.1 M KOH	N_2_/H_2_O	3.47 × 10^−12^	1.96	[48]
20	Nano-Fe_2_O_3_	Pt	Nafion 115/0.5 M KOH	N_2_/H_2_O	1.49 × 10^−12^	-	[30]
90	MOF (Fe)	Pt	Nafion 117/2 M KOH	N_2_/H_2_O	2.12 × 10^−9^	1.43	[49]
90	MOF (Co)	Pt	Nafion 117/2 M KOH	N_2_/H_2_O	1.64 × 10^−9^	1.06	[49]
90	MOF (Cu)	Pt	Nafion 117/2 M KOH	N_2_/H_2_O	1.24 × 10^−9^	0.96	[49]
90	MOF (Fe)	Pt	Nafion 117/2 M KOH	N_2_(Air)/H_2_O	1.52 × 10^−9^	0.88	[49]
AT	Rh NNs	Carbon rod	Nafion211/0.1 M KOH	N_2_/H_2_O	6.24 × 10^−9^	0.7	[50]
AT	Carbon nanospikes	Pt	Membrane/0.25 M LiClO_4_	N_2_/H_2_O	1.59 × 10^−9^	11.56	[51]
AT	Ni	Pt	2-propanol: 0.01 M H_2_SO_4_ (9:1v/v)	N_2_/H_2_O	1.54 × 10^−11^	0.89	[52]
25	Ni	GC	CMX/0.1 M LiCl in EDA	N_2_/H_2_O (0.05 M H_2_SO_4)_	3.58 × 10^−11^	17.2	[53]
AT	α-Fe/Fe_3_O_4_	Pt	[C_4_mpyr][eFAP] FPEE mix	N_2_/H_2_O	2.35 × 10^−11^	32	[54]
AT	Fe-Stainless Steel mesh	Pt	[P_6,6,6,14_][eFAP] ionic liquid	N_2_/H_2_O	2.04 × 10^−11^	46	[55]
AT	Fe-Stainless Steel mesh	Pt	[C_4_mpyr][eFAP] ionic liquid	N_2_/H_2_O	2.2 × 10^−11^	35	[55]
AT	Fe-Fluorine doped tin oxide glass	Pt	[C_4_mpyr][eFAP] ionic liquid	N_2_/H_2_O	6.5 × 10^−12^	38	[55]
AT	Fe-Fluorine doped tin oxide glass	Pt	[P_6,6,6,14_][eFAP] ionic liquid	N_2_/H_2_O	6.5 × 10^−12^	60	[55]
AT	Fe-Nickel foam	Pt	[P_6,6,6,14_][eFAP] ionic liquid	N_2_/H_2_O	1.88 × 10^−11^	21	[55]
RT	Ag-Au/ZIF	Pt	THF-based electrolyte	N_2_/H_2_O	1 × 10^−11^	18 ± 4	[56]
AT	Pt/C	Pt/C	AEM	N_2_-H_2_O	1.96 × 10^−11^	1.73	[57]
65	Fe_2_O_3_/CP	Ti/IrO_2_	FAA-3 Fumatech (AEM)	N_2_-H_2_O	1.91 × 10^−13^	0.044	[48]

* Similar values obtained with Ar in place of N_2_.

## Abbreviations

AC	Activated carbon
AEM	Anion exchange membrane
AT	Ambient temperature
CC	Carbon cloth
CNT	Carbon nanotubes
CP	Carbon paper
DFT	Density functional theory
EDA	Ethylene diamine
FE	Faradaic efficiency
PEBCD	Poly(*N*-ethyl-benzene-1,2,4,5-tetracarboxylic diimide)
GC	Glassy carbon
H-B	Haber-Bosch process
HNC	Hollow nanocages
MOF	Metal-organic-framework
NCM	N-doped nanoporous graphitic carbon membrane
NPC	Ν-doped porous carbon
NRR	Nitrogen reduction reaction
OCV	Open circuit voltage
PBS	Phosphate buffer solution
RGO	Reduced graphite oxide
RT	Room temperature
SSAS	Solid state ammonia synthesis
THF	Tetrahydrofuran solution
ZIF	Zeolitic imidazolate framework
